# Application of dental pulp stem cells in oral maxillofacial tissue engineering

**DOI:** 10.7150/ijms.68494

**Published:** 2022-01-11

**Authors:** Peng Liu, Yingxin Zhang, Yujie Ma, Shuang Tan, Bingyi Ren, Shitao Liu, HuanYan Dai, Zhimin Xu

**Affiliations:** 1 Hospital of Stomatology, Jilin University, Changchun 130021, China.; 2 Department of Oral Emergency, Hospital of Stomatology, Jilin University, Changchun 130021, China.; 3 Department of Oral and Maxillofacial Surgery, School and Hospital of Stomatology, Jilin University, Changchun 130021, China.

**Keywords:** Dental pulp stem cells, periodontal disease, tissue-engineered, osteogenic

## Abstract

In the maxillofacial area, soft and hard tissue abnormalities are caused by trauma, tumors, infection, and other causes that expose the maxillofacial region to the surface of the human body. Patients' normal physiological function and appearance are interfered with, and their mental health is adversely impacted, reducing their overall life quality. The pursuit of appropriate medical treatments to correct these abnormalities is thus vital. Autologous stem cell regeneration technology mainly focused on tissues has lately emerged as a significant problem in the medical community. Because of the capacity of dental pulp stem cells (DPSCs) to self-renew, the use of DPSCs from the human pulp tissues of deciduous teeth or permanent teeth has gained popularity among scientists as a stem cell-based therapy option. Aside from that, they are simple to extract and have minimal immunogenicity. As a result, bone tissue engineering may be a critical component in treating maxillofacial and periodontal bone abnormalities. DPSCs activity in maxillofacial and periodontal tissue-engineered bone tissue was investigated in this research.

## Introduction

Oral and maxillofacial injuries are classified as hard tissue and soft tissue injuries. Among the most often utilized treatment techniques for oral and maxillofacial jaw fractures include reduction and fixation and autologous/allograft bone defect repair if substantial bone loss has occurred. Nonetheless, autologous bone transplantation may result in additional surgical stress, microbial allograft contamination, delayed healing, and cellular and humoral immunity; the two techniques have their benefits and drawbacks 1, 2. Current treatment options for oral and maxillofacial soft tissue injuries mainly focus on infection control, tissue/blood vessel reduction, and suture insertion. Depending on the degree of the tissue defect, skin grafting or skin flaps may be used to repair it; however, this may result in donor site tissue injury, and it is unknown if the skin flap will survive and restore sensory function. As a result, existing healing methods have limitations, and it is difficult to completely repair the damaged part of the patient's body after the operation 3, 4.

Tissue engineering technologies based on allogeneic stem cells have been steadily developed because they do not involve ethical controversy or cause immunogenicity. The wait for a donor causes no diagnostic delay. Such stem cells in humans exhibit two characteristics: self-renewal and the capacity to undergo multidirectional differentiation in adulthood 5, 6. Unlike transplanted bone, tissue-engineered bone has low antigenicity, plastic properties, and an ideal shape and involves a short operating time, among other benefits 7, 8.

Since mesenchymal stem cells (MSCs) have good proliferative and multidirectional differentiation capacities, they have attracted much attention in bone tissue engineering science 9. Indeed, MSCs are the most used seed cells in tissue repair, as they exhibit direct osteogenic differentiation and low immune rejection. At the same time, they can promote tissue regeneration by adjusting the anterior mesenchyme of the bone marrow, skin, teeth, and other parts of the human body from which stem cells have been successfully isolated^10^, ^11^. Furthermore, MSCs have the advantage of their differentiation into various human cell types or tissues *in vivo* or *in vitro*, serving as a source of cells for organ repair and self-somatic cells. Among MSCs, human dental pulp stem cells (hDPSCs) are deciduous teeth and permanent teeth with active self-renewal and multidirectional differentiation potential 12, 13.

Because of their many attractive qualities (easy access to the collection site, low morbidity, highly efficient extraction and processing, osteogenic differentiation ability, and high interactivity with scaffold materials), DPSCs have become a research hot spot. This review aimed to report recent advances in the engineering of dental pulp stem cells in the oral and maxillofacial fields and analyze periodontal tissue applications in the bone.

## Biological characteristics of DPSCs

### Diversification and strong proliferative potential

The biological characteristics of DPSCs and their multidirectional differentiation potential and immunomodulatory function are similar to those of bone marrow mesenchymal stem cells (BMSCs). However, DPSCs have essential phosphoric acid ALP activity more significant than that in BMSCs 14, 15. A study has shown that DPSCs cultured *in vitro* are fusiform and can differentiate into osteoblasts, adipocytes, hair follicle cells, corneal epithelial cells, nerve cells, and melanocytes under appropriate induction conditions 16. Although both DPSCs and BMMSCs can differentiate in the direction of bone, cartilage, muscle, fat, and nerve, DPSCs show higher differentiation ability, and their proliferation rate is 30-50 times that of BMMSCs. This suggests that DPSCs will provide a new development direction for tissue repair and regenerative medicine 17.

### Immune regulation ability

Fas ligand (FasL) is a transmembrane protein required for the Fas apoptotic pathway to function properly. Elimination of FasL from DPSCs using siRNA technology decreases its capacity to trigger T cell death and anti-inflammatory effectiveness, indicating that FasL regulates DPSCs' immunomodulatory ability. However, the amount of FasL expression did not affect the proliferation rate or capacity of DPSCs to differentiate in several directions 18. DPSCs have a substantial inhibitory effect on T Helper 17 (Th17) cells and have been shown to cure systemic lupus erythematosus (SLE)-associated dysfunction after transplantation. Also, DPSCs suppressed lymphocyte proliferation through the secretion of transforming growth factor-1 (TGF-1) 19. Likewise, transplantation of DPSCs into ischemia and hypoxic mice models has been shown to induce an anti-inflammatory response and facilitate tissue healing 20.

Similarly, LPS may significantly increase interleukin-8 (IL-8) expression in DPSCs. IL-8 is a chemokine cytokine with chemotactic properties toward neutrophils, which plays a critical role in controlling the inflammatory response. If differentiated DPSCs retain the same immunomodulatory capacity as undifferentiated DPSCs, they may be utilized as a stem cell resource to treat immunological disorders by modulating immune properties 21.

### Paracrine action

DPSCs may produce a variety of cytokines in a paracrine manner, including the chemokine stromal cell-derived factor-1, brain-derived neurotrophic factor, ciliary neurotrophic factor, nerve growth factor, vascular endothelial growth factor, granulocyte-colony stimulation factor, and stem cell factor are all growth factors. Those could stimulate angiogenesis, inhibit apoptosis, and preserve regenerated tissue 22, 23. Indeed, in an ischemia animal model, it was stated that DPSCs had better cytoprotective effects on astrocytes. Additionally, DPSCs may produce functional neovascularization in mice models of hind limb ischemia 24. Apart from transforming into osteoblasts, DPSCs may also promote bone tissue regeneration by releasing paracrine substances such as various growth factors, which these cells release in a conditioned culture media 25.

The pulp is a loose connective tissue with repair and regeneration capabilities. Mild inflammation can stimulate DPSCs retained in the pulp to migrate to the injured site and then differentiate into odontoblast cells and participate in repairing the dentin-pulp complex 26. Therefore, promoting pulp tissue regeneration under the inflammatory microenvironment has become a research hotspot. Likewise, co-cultured of DPSCs with concentrated growth factor (CGF), Lipopolysaccharide (LPS), and CGF+LPS, respectively, could promote the regeneration of dentin-pulp complex of immature beagle teeth, which suggested that CGF might be a substitute biomaterial for pulp regeneration therapy 27.

## Differication of DPSCs

Recently, a large amount of research has been published on the function of miRNA in the differentiation of DPSCs. Indeed, miRNA promotes the differentiation of DPSCs into various cell types and the performance of corresponding functions *in vitro* by directly or indirectly interconnecting with different signaling pathways, demonstrating efficient regulatory effects and demonstrating the distinct advantages of DPSCs when compared to other stem cells. It introduces a novel concept and technique for using DPSCs as seed cells in the process of tissue regeneration. Even though it has significant utility and application potential in tissue engineering and regenerative medicine, its use in live tissue or organ regeneration has only been documented in a few cases. Despite this, the therapeutic use of DPSCs may encounter the following difficulties:

- The techniques for cryopreservation and preserving the stem cell activity and properties of DPSCs are not yet fully developed.
- There is still a long way to go to identify highly targeted miRNA at various phases of DPSC differentiation and maturation. It has been shown that miRNA controls DPSC differentiation and performs related activities, which may be used in tissue engineering and regenerative medicine to benefit the patient.

In brief, Table 1 summarizes the regulation of microRNAs in the differentiation of DPSCs.

## Application of dental pulp stem cells in tissue engineering

### The tooth tissue area and periodontal tissue regeneration

Periodontal disease is a chronic infectious disease that causes tooth loss; another major cause of tooth loss is alveolar bone resorption and severe periodontal tissue destruction in the late stage of periodontal disease 25. Recently, researchers used homologous dental pulp stem cells and periodontal ligament stem cells to treat periodontitis in a miniature pig surgical model. They found that growth factors and morphological factors are essential in periodontal tissue engineering; both were mainly used to improve the proliferation and differentiation of stem cells during synthesis and the secretion of a mineral matrix. Indeed, multiple growth factors, such as enamel matrix derivatives, have been identified and successfully supported periodontal regeneration 38-40. Regardless, it was reported that treating titanium implants' surfaces with specific microRNAs might speed up the osseointegration of human dental stem cells. Also, ion blasting and acid etching of surfaces encouraged osteoblast formation. This discovery might increase the therapeutic effectiveness of osteoblasts. Therefore dental pulp stem cells can repair the pulp-dentin complex. Tissue engineering may repair injured pulp and regenerate tooth and periodontal tissues, offering fresh hope 41, 42.

### The bone tissue regeneration field

*In vitro* studies showed that the injection of dental pulp stem cells with gelatine scaffolds could ectopically shape bone structures, and seed cells from bone tissue can be used as advanced stem cells, opening a new direction in bone tissue engineering 43. It was reported that SOX2 regulates osteogenic genes and bone morphogenetic proteins, thus promoting the osteoblast differentiation of dental pulp stem cells 27, 44. Yang et al. 45 implanted DPSCs and BMP-2 into naked mice and discovered that mineralized tissues formed 12 weeks later. Similarly, it was discovered that BMP-7 could be used *in vitro* for DPSCs at concentrations ranging from 25 to 100 g/L with a dose-dependent formula to regulate dentine sialophosphorin, osteocalcin, dentine basal albumin-1 and alkaline phosphatase (ALP), Runt phase transcription factor 2 expression, and to promote dentin differentiation via activation of the Smad5 channel 46. Similarly, when BMP-2 was paired with BMP-7 or other growth factors, such as mineralized fluid or vascular endothelial growth factors, the effectiveness of initiating DPSC osteogenesis toward differentiation was significantly increased 47.On the other hand, when DPSCs were treated with a single or combination mineralized solution of BMP-2, there was no evidence of osteogenic factor expression in the single treatment group. Still, there was evidence of osteogenic marker gene expression in the combined induction group 48.

Likewise, Xia et al. 49 reported a new phosphoric acid binder containing gold nanoparticles (GNPs) enhanced dental stem cell adhesion to phosphoric acid. Junction agent activity increased cell adhesion, proliferation, and the osteogenic differentiation force (2-3 times within 14 days). Additionally, Paduano et al. 50 evaluated the differentiation of dental pulp cells on hydrogel scaffolds and dental pulp cells seeded on collagen I into the tooth. They found that when dental pulp stem cells were in a hydrogel scaffold, significantly more DSPP, DMP-1, and MEPE gene mRNA expression was observed to increase under culture.

### Blood vessel construction

The pulp is a highly vascularized and innervated tissue containing a group of stem cells with the potential for multidirectional differentiation. Because of their vascular characteristics, dental pulp stem cells are a new approach to treat diseases related to small blood vessels. Teeth can be collected and exposed to a wide range of growth factors, including those from the conditioned medium of myeloid stem cells, neurotrophic factors, nerve growth factors, vascular endothelial growth factors, and glial cell-derived neurotrophic factors 51. Yamaguchi et al. 52 used a mouse model to study human dental pulp stem cells' therapeutic effect on induced myocardial infarction. They found that the site of dead tissue shrank, accompanied by the formation of new blood vessels, which may be due to supplemental endothelial growth factor. Likewise, Silva et al. [53]evaluated the effects of lipoprotein receptor-associated protein 6 (LRP6) on endothelial differentiation and the capillary formation LRP6-mediated signaling pathway contributes to the vascular differentiation of DPSCs. Due to their vascular formation characteristics, dental pulp stem cells may be a new way to treat vascular insufficiency in disease.

### Corneal reconstruction

Corneal blindness is a severe eye condition. At the moment, corneal allograft transplantation is the primary technique of therapy, but donor shortages and immune rejection reactions restrict its practical use 54. Tissue engineering of the cornea is a popular subject in ophthalmology, and appropriate seed cells are critical 55. Research suggested that DPSCs may enhance corneal epithelial cell development and regeneration, prevent corneal conjunctiva, and preserve corneal clarity 56. Furthermore, it was reported that after *in vitro* differentiation, DPSCs could develop into corneal stromal cells, corneal protein, and keratin sulfate, suggesting that DPSCs can differentiate into corneal stromal cells 57. Gomes et al. 58 established dental pulp-based stem cells from a tissue engineering component transplanted into a rabbit with a corneal defect. They found that dental pulp-based stem cells could promote corneal epithelial reconstruction. Besides, studies have shown that photoreceptors transplanted into the outer retinal nuclear layer could restore visual function 59. However, it is not clinically feasible to acquire many photoreceptors from the eyes of young donors 60. These findings demonstrate that DPSCs may have therapeutic use in corneal regeneration tissue engineering research, but further study is required to elucidate the mechanism by which DPSCs promote corneal regeneration.

### Cartilage formation

Some populations of DPSCs cells, extracted from the pulp, can express factors characteristic of bone, such as type II collagen and chitosan, and approximately 30% of those can be transformed into chondrocytes. Moreover, dental pulp stem cells at the early stage of culture could be differentiated into dentin, bone, and cartilage structures. Still, these cells at the late stage of culture could be differentiated into osteoblasts 61, 62. The differentiation of dental pulp stem cells into chondrocytes suggests that the use of dental pulp stem cells may be an effective measure in tissue engineering to treat cartilage lesions 62.

### Blood vessels and muscles

Since the face is densely packed and has adequate blood circulation during a maxillofacial injury, the facial blood vessels are particularly vulnerable to damage or fracture. If you can rapidly restore blood flow to the tissue, it will aid in the organization of oxygen in the blood vessels and the delivery of nutrients, allowing the face damage to heal more quickly. There are many methods in which hDPSCS may aid in the repair and regeneration of blood vessels, including the following:

- In the presence of vascular endothelial growth factor (VEGF), DPSCs may develop into endodermal cells 63, 64.
- DPSCs release VEGF and other substances to promote vascularization and engage in vascular remodeling as perivascular cells 65, 66.
- DPSCs repair and restore neovascularization by promoting endothelial cell migration 67.

At the same time, identifying the stimulating factors and scaffolds that influence the ability of DPSCs to repair blood vessels has been the primary focus of study in this area. Among them, VEGF is a significant stimulating agent that encourages DPSCs to repair blood vessels, as described below: In a co-culture with VEGF and 5 percent concentrated growth factor, endothelial cells generated from DPSCs were shown to develop mature tubule-like structures on the basement membrane 68. Not only may VEGF be used as a stimulator to induce vascularization, but it can also be used as a marker of vascular differentiation to assess the effects of many other stimuli 27. Whether or not certain variables may enhance the vascular differentiation potential of DPSCs is still being investigated. Overexpression of IGFBP5 in DPSCs has been shown to increase VEGF expression, platelet-derived growth factor A, and other vascular differentiation markers, indicating that this situation is favorable to vascular remodeling DPSCs 69.

Furthermore, DPSCs have been demonstrated to be capable of myogenic differentiation, and microRNAs (miRNAs) have been shown to play a critical part in this process 70. The binding of DPSCs to the receptor's muscle fibers and blood vessels has been shown *in vivo* after being injected into the skeletal muscles of malnourished mice, resulting in fibrosis, reduced collagen content, and increased cross-sectional area of the muscle fibers^71^. However, because of the anatomical peculiarities of the maxillofacial blood tube and muscle, more debate on how to use DPSCs to maxillofacial blood vessel and muscle damage is required.

Duchenne muscular dystrophy (DMD) is severe muscular dystrophy caused by a deficiency of functioning structural proteins in the muscles. Patients often die in their early twenties due to heart and lung muscle-weakening 72. DPSCs may ameliorate the pathologic state of dystrophic skeletal muscle tissue in DMD mice by decreasing fibrosis and increasing angiogenesis, opening the door for future research on the mechanism by which DPSCs are more efficiently utilized in muscle repair and regeneration 73.

### Nerve repair

The implantation of mesenchymal stem cells derived from the pulp of human deciduous teeth could effectively assist in wound recovery 74. Moreover, due to their dynamic differentiation, dental pulp stem cells have paracrine effects 75. Besides, the IGF1 receptor, a pluripotent cell marker in embryonic stem cells, indicating self-renewal and differentiation potential, is expressed in the dental pulp. Dental pulp stem cells secrete IGF1, which interacts with IGF1 receptors to maintain self-renewal and proliferation potential through the autocrine signaling pathway. Likewise, stereotactic implantation of the IGF1 receptor in oxygen-ischemia rats could enhance the expression of the apoptotic protein Bcl-2 and improve neural plasticity 76.

DPSCs derived from various cranial neural crest cell lineages express several factors that promote nerve and axon regeneration, apart from their significant neural differentiation potential. DPSCs have been shown to exhibit the neural crest cell markers CD271 and SOX10, which may be used to stimulate the production of Schwann cells, which play a critical role in peripheral nerve repair 77. Under certain conditions, DPSCs may be induced to differentiate into neuronal cell lines and can also stimulate cell self-repair through the paracrine signaling pathway. While co-cultured with the hippocampus, it was found that DPSCs could produce the neurocytokine BDNF in the Golgi body but not of expressing neuronal markers or exhibiting neuronal cell morphology 78. DPSCs were implanted into rats with a completely severed spinal cord, and it was discovered that they could prevent meridians from undergoing apoptosis as a result of spinal cord injury. As well, directly inhibit the generation of numerous neuronal growth inhibitors via the two mechanisms described above and even replace inactivated nerve cells by differentiating into mature oligodendrocytes 79.

**Figure 1:**
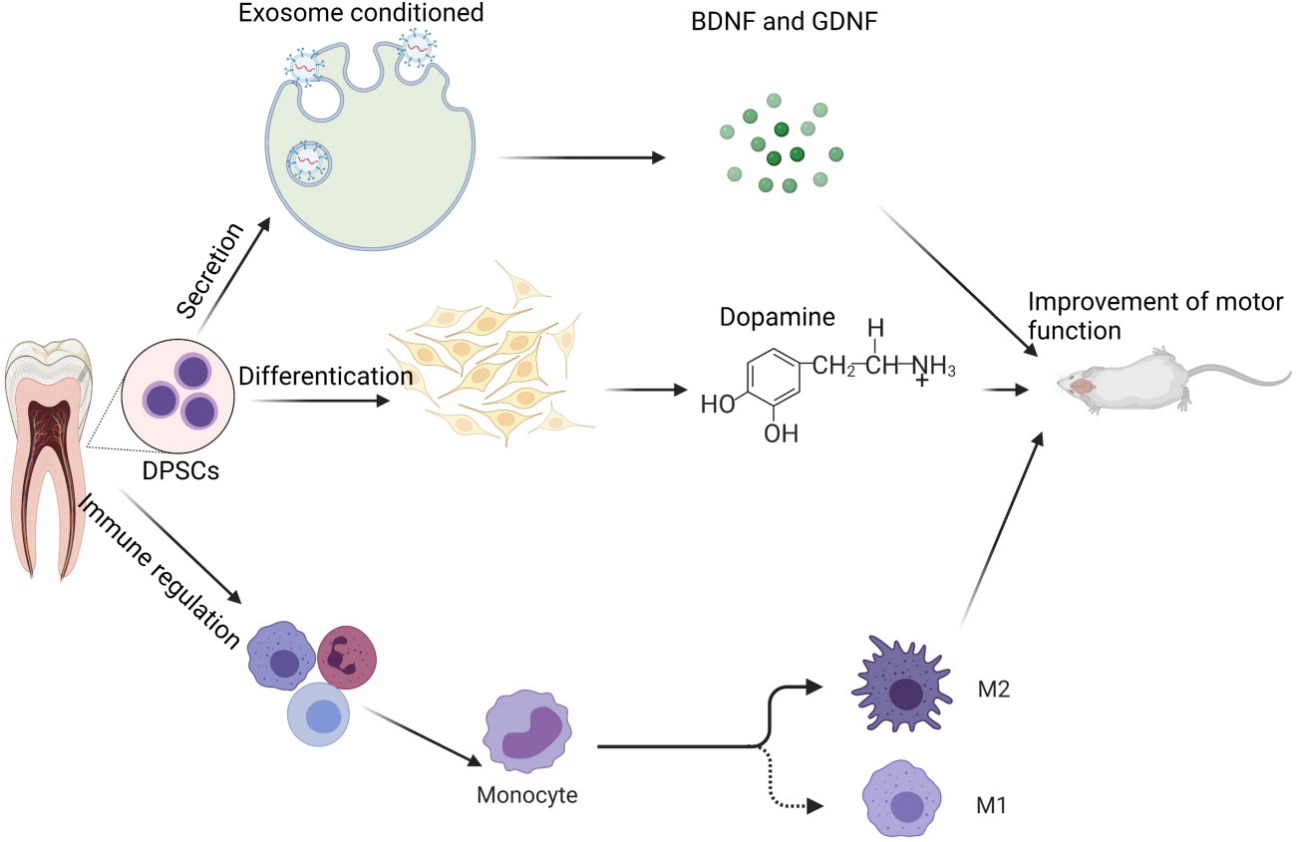
** Potential mechanism of DPSCs in neurological diseases:** In the therapy of neurologic illness, DPSCs may be classified into three types of mechanisms: 1) improving brain function via the secretory route; 2) differentiation of new dopaminergic neuron (like) cells; and 3) immune control, which reduces the inflammatory response. Note: Glial cell-derived neurotrophic factor (GDNF) and the and brain-derived neurotrophic factor (BDNF)

Researchers are still looking for appropriate growth factors and scaffold materials to improve DPSCs' ability to repair damaged nerves and study the mechanism by which DPSCs repair damaged nerves. According to studies, DPSCs producing high amounts of recombinant human insulin-like growth factor-binding protein 5 (IGFBP5) exhibit greater NCAM, TH, and other neurogenic markers. IGFBP5 has the ability to enhance the direct differentiation of odontogenic stem cells into meridians in some ways by increasing the size and amount of nerve spheres generated by DPSCs 80. DPSCs need epidermal growth (EGF) and basic fibroblast growth factors (BFGF). EGF can develop into neuron-like cells in response to BFGF stimulation 81. Combining DPSCs, gelatin methacrylate hydrogel, and BFGF has been shown to heal a 15-mm sciatic nerve lesion in rats. Not only may human DPSCs develop into neural-like cells in rats, but they can also stimulate nerve regeneration in rats 82. Some researchers loaded DPSCs with a siloxane-controlled scaffold wrapped in type I collagen and then transplanted them into the buccal notch of Louis mice's facial nerve. They discovered that DPSCs tubes could effectively restore facial nerve defects in function and electrophysiology, comparable to the nerve defects restored by autologous transplantation in mice 83. Clinically, the inferior alveolar nerve tube is often injured due to trauma or surgery, significantly impairing patients' quality of life. Thus, the use of DPSCs as seed cells to heal the inferior maxillary nerve/inferior alveolar nerve damage is an area that warrants further investigation 84. Figure 1 below describes the mechanism of DPSCs protection on neural.

### Temporomandibular joint regeneration

When it comes to oral mastication, articulation, and speech, among other things, the temporomandibular joint (TMJ) is critical. The articular disc is the most susceptible part of the TMJ, and the articular disc is mainly comprised of cartilage tissue, making it the most vulnerable part of the joint. As a result, DPSCs have been utilized to differentiate chondrocytes to restore the cartilage tissue that has been injured 85. According to a morphological examination of the cells, it was discovered that alkaline phosphatase activity in DPSCs increased on day 14 after the induction of chondrogenesis when they were grown under chondrogenic conditions. On day 21, proteoglycans were discovered in the DPSCs 86. Furthermore, type I and type II collagen were strongly expressed in both periods, and DPSCs could differentiate into chondrocytes 87.

DPSCs may differentiate into cartilage using various scaffold materials, which are now the subject of a significant number of studies 88. Indeed, researchers discovered that poly(ethylene glycol dimethacrylate-methacrylate anhydride gelatin-hyaluronic acid hydrogel nanostructure supports (PEG-GELma-HA) hydrogel nanostructure supports can effectively stimulate chondrogenic differentiation of DPSCs^89^. Likewise, DPSCs cultivated on calcium chloride/glutaraldehyde (chitosan/alginate, Ch/Alg) scaffold found that it not only allowed for the attachment and proliferation of DPSC cells for an extended period but it also allowed them to grow indefinitely. According to the results obtained after 4-8 weeks, a significant amount of fibrous cartilage tissue had formed on the scaffold. Furthermore, the mechanical properties of the scaffold loaded with DPSCs were significantly improved compared to those of the cell-free scaffold, and the relevant data were comparable to those of a natural TMI disc 90.

## Application of DPSCs in maxillofacial tissue-engineered bone

The maxilla and mandible are two of the 14 bones that comprise the maxillofacial region. DPSCs have been demonstrated in previous studies to have osteogenic potential and may assist in repairing bone tissue. At the moment, the majority of research on DPSCs repairing maxillofacial bone tissue is still in animal trials, and there is insufficient clinical experimental evidence to justify their usage. DPSCs have been found to promote the repair of skull abnormalities in rats when used with a strong collagen scaffold. The new bone generated is membrane-internalized, identical to the osteogenesis of the skull 91.

The progressive loss of jaw bone tissue and periodontal tissue inflammation are the primary causes of maxillofacial bone tissue abnormalities in the maxillofacial area. SHEDs may help to reduce periodontal inflammation by polarizing M2 macrophages. Promote periodontal tissue regeneration in the context of inflammation 92.

There have been many studies on the application of DPSCs to bone tissue regeneration, and most of their results showed that DPSCs are beneficial for bone tissue deficiency, lesion repair, and healing. Nevertheless, relatively few studies have been on the woven bone from the oral, maxillofacial, and periodontal fields 93**.** Periodontal ligament stem (PDL), namely undifferentiated mesenchymal cells in the Periodontal membrane, are a class of cells with heterogeneity and differentiation potential, which have the characteristics of adult stem cells and are essential cells for Periodontal tissue regeneration 94. Regardless, Khorsand et al. 95 developed a canine periodontitis model in which bone powder was mixed with autologous canine DPSCs and subsequently used to repair the damaged bone. After eight weeks, the periodontal membrane's structural regeneration and bone cementum were detected. Histomorphometric investigations revealed that the quantity of regenerated cementum and periodontal ligament was substantially more significant in the test groups than in the control groups. A biocomplex composed of DPSCs and Bio-Oss might be promising for periodontal tissue regeneration. Also, with 3D printing technology, Lee et al. 96 fabricated polycarpic acid with lactone/hydroxyapatite laminate materials with micropores of different sizes separated the layers containing connective tissue growth factor and compounded the material with PGLA nanospheres containing BMP-2. Used to carry DPSCs, the material was implanted under the skin of nude mice, and bone samples with periodontal membranoid tissue dentine/cementitious tissue formation were found. Interestingly, similar to those from the physiological periodontal membrane, collagen fiber bundles from the new periodontal membrane-like tissue extended into the hard tissue.

Aimetti et al. 97 isolated DPSCs from a patient's third molar and combined them with the collagen sponge scaffold. The periodontal pocket of the second premolar had filled after one year, and the depth of periodontal exploration and loss of attachment had decreased by 6 mm. Likewise, Brunelli et al. 98 performed maxillary sinus extraction before surgery. The third molar pulp was mechanically ground; the resultant cell suspension poured into the collagen sponge and used to fill a defect in the maxillary sinus floor, followed by immediate implantation. Four months later, X-ray and CT results showed evident elevation of the maxillary sinus floor, and much new bone had formed.

**Figure 2:**
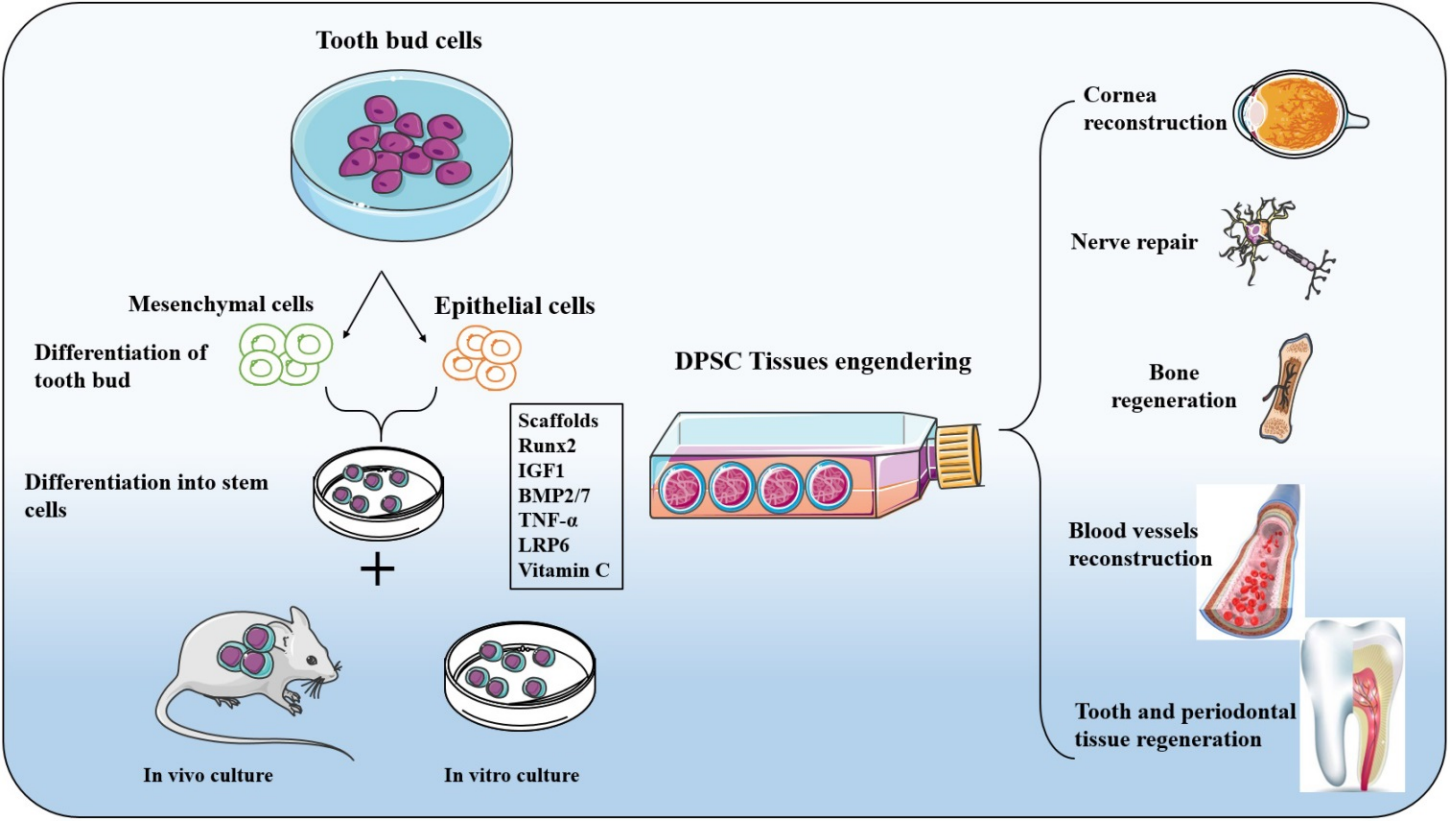
** Differentiation and tissue engineering application of DPSCs cells:** Adult bone remodelling is continuously remodelled using osteoclast, osteoblasts, and simultaneous osteoclasts. Stem cells and progenitor cells located in the endometrium and periosteum have limited ability to regenerate and need surgical intervention. The use of biomaterials or bone grafting that mimics bone structure is an essential way to solve bone loss. DPSCs, after differentiation, show the same protein components in mineralized tissues such as osteocalcin, alkaline phosphatase, and osteopontin so that DPSCs can be an excellent source of regenerative osteoblasts and chondroblasts for alveolar and maxillofacial bone. DPSCs originated from nerve ridges and migrated and differentiated to participate in morphogenesis. Therefore, DPSCs have been more widely used in surgical repair and regeneration, resulting in cranial and facial structures, including muscles, ligaments, cartilage, bones, periodontal membranes, and teeth. The expression of these osteogenic markers is related to osteogenic and odontogenic induction drugs. DPSCs can differentiate into functional osteoclasts in vitro, and they have also been found to produce an extracellular mineralized matrix.

On a high concentration hydroxyapatite-collagen scaffold, the ability of DPSCs to differentiate into osteoblasts was enhanced, and DPSCs covered the entire surface of the scaffold and formed continuous cell layers under scanning electron microscopy, in addition to filamentous pseudopod expansion 49, 99. As a consequence of the continuous development of nanomaterials, researchers have used nanoparticles, nanofibers, and even a mixture of nanotubes and gels as support materials to promote DPSC adherence, proliferation, and bone regeneration 100, 101. The proliferation and differentiation of DPSCs were guided by changing the size or density of nanoparticles on the scaffold 102. To avoid future harm or disease-free problems in patients, DPSCs will gradually replace existing autologous/allogeneic bone transplants. They will play a critical role in repairing maxillofacial bone abnormalities, aided by advances in materials science. Figure 2 below illustrates the differentiation and opportunity of tissue engineering application of DPSCs cells.

## Discussion and prospects

DPSCs are taken mostly from naturally replaced deciduous teeth, third molars, orthodontic teeth, and teeth that must be pulled for other reasons. As a source of easy-to-obtain seed cells for autologous transplantation, DPSCs offer several benefits as tissue engineering seed cells, making them an attractive candidate for clinical use. Although many advances in the study and use of DPSCs have been achieved, they have mostly been confined to animal models. There are still many obstacles to overcome before DPSCs can be effectively used for therapeutic tissue regeneration. For instance, the precise surface markers of DPSCs are unknown. It proved challenging to identify in pulp tissue precisely. Differentiation's regulating mechanism remains unknown. First, there are methods to acquire stable and dependable seed cell sources. *In vitro* expanded dental pulp stem cells take a long time. Which stage of dental pulp stem cell differentiation is best for transplanting. Second, *in vitro* tissue manufactured teeth and other cell treatments are hindered by the lack of breakthroughs in critical technologies such as three-dimensional cell culture, biological scaffold materials, and physiological microenvironment modelling. As tissue engineering research progresses, it is expected that all of these issues will be resolved in the near future. This will allow proper tissue and organ regeneration and the most favourable conditions for treating oral and systemic diseases.

Clinically, many patients do not exhibit the conditions for DPSC transplantation *in vivo*, in contrast to homologous allogeneic DPSCs, which could be applied to more cases by, for example, building a cell bank. At present, DPSCs and stem cells from human foliated deciduous teeth are available, and dental stem cell banks have been set up for future clinical needs. The characteristics of bone defects should be further studied, especially establishing significant bone defects, to explore the best strategy for bone tissue engineering. However, it is believed that with the advancement and development of tissue engineering technology, the integration of life science, engineering, materials science, and other related disciplines, as well as the unwavering efforts of scientific researchers. These issues will be discussed in greater detail and eventually resolved. The research of DPSCs is progressing at a rapid pace, and genuine tissue regeneration is becoming a reality.

In recent years, stomatology has focused on pulp regeneration. Its exact shape and structure promote seed cell adhesion and growth factor release. Indeed, researchers 3D printed a “pulp complex” using a tissue engineering scaffold to investigate pulp regeneration. This article explores the use of 3D printing in pulp regeneration. According to the literature review, the scaffold material, seed cells, and growth factors in 3D printed “pulp complexes” are crucial in pulp regeneration research. The scaffold material may transport seed cells and growth factors to a suitable microenvironment. Standard seed cells for pulp regeneration include pulp stem cells, apical papilla stem cells, and human deciduous tooth pulp stem cells. Growth factors may help differentiate pulp tissue and regenerate pulp blood vessels, facilitating pulp regeneration. Currently, the 3D printing “pulp complex” in the study of dental pulp regeneration has made some progress, but providing oxygen and nutrients to root canal cells is still a considerable challenge that requires more exploration and research.

3D stem cell culture has great potential for tissue and organ regeneration, allowing the creation of *in vitro* bone, liver, heart, and brain models. 3D stem cell cultivation may one day be used to create pulp tissue and disease models *in vitro*. However, 3D stem cell culture still has unresolved challenges. As a result of the delicate culture processes and unexpected culture yields, it is now challenging to popularize 103. However, modern imaging technology, detection equipment, and detection processes must examine the complicated shape and function of stem cells or microtissues 104. The utilization of allogeneic stem cells and mass manufacturing, freezing, and resuscitation of stem cells are difficulties to overcome throughout the shift from fundamental research to clinical practice.

## Figures and Tables

**Table 1:** Regulation of microRNAs in the differentiation of DPSCs.

DPSCs	miRNA	Mechanisms	References
**Odontogenic differentiation**		Inhibit Wnt1 signaling pathways	28
	MiR-140-5P	Inhibit TLR4	29
	MiR-143-3P	Inhibit RANK	30
	MiR-125a-3P	Promote Fyn	31
**Osteogenic differentiation**	MiR-143	Inhibit NF-κB via targeting TNF-α	32
	MiR-146a-5P	Regulate STRO-1^+^ through NOTCH1 signaling pathways	33
	MiR-496	Inhibit CirRNA12453/β-Catenin signaling pathways	34
**Vascular differentiation**	MiR215/miRNA219a-1-3P	Downregulate HspB8	35
	MiR411	Inhibit HIF-1α and promote autophagy	36
	MiR-140-5p	Inhibit Wnt1 signaling pathways	28
**Neurogenic differentiation**	MiR-454	Inhibit ephrin receptor B4 promotes the proliferation and invasion of trophoblast cells	37

## References

[1] Goh EZ, Beech N, Johnson NR (2021). Traumatic maxillofacial and brain injuries: a systematic review. Int J Oral Maxillofac Surg.

[2] Zhang Q, Wu W, Qian C, Xiao W, Zhu H, Guo J (2019). Advanced biomaterials for repairing and reconstruction of mandibular defects. Mater Sci Eng C Mater Biol Appl.

[3] Liu Y, Sun X, Yu J, Wang J, Zhai P, Chen S (2019). Platelet-Rich Fibrin as a Bone Graft Material in Oral and Maxillofacial Bone Regeneration: Classification and Summary for Better Application. Biomed Res Int.

[4] Greenberg AM (2015). Digital technologies for dental implant treatment planning and guided surgery. Oral Maxillofac Surg Clin North Am.

[5] Shang F, Yu Y, Liu S, Ming L, Zhang Y, Zhou Z (2021). Advancing application of mesenchymal stem cell-based bone tissue regeneration. Bioact Mater.

[6] Sui BD, Hu CH, Liu AQ, Zheng CX, Xuan K, Jin Y (2019). Stem cell-based bone regeneration in diseased microenvironments: Challenges and solutions. Biomaterials.

[7] Khachatryan L, Khachatryan G, Hakobyan G (2018). The Treatment of Lower Jaw Defects Using Vascularized Fibula Graft and Dental Implants. J Craniofac Surg.

[8] Liang F, Leland H, Jedrzejewski B, Auslander A, Maniskas S, Swanson J (2018). Alternatives to Autologous Bone Graft in Alveolar Cleft Reconstruction: The State of Alveolar Tissue Engineering. J Craniofac Surg.

[9] Buduru SD, Gulei D, Zimta AA, Tigu AB, Cenariu D, Berindan-Neagoe I (2019). The Potential of Different Origin Stem Cells in Modulating Oral Bone Regeneration Processes. Cells.

[10] Ansari S, Seagroves JT, Chen C, Shah K, Aghaloo T, Wu BM (2017). Dental and orofacial mesenchymal stem cells in craniofacial regeneration: The prosthodontist's point of view. J Prosthet Dent.

[11] Hu L, Yin C, Zhao F, Ali A, Ma J, Qian A (2018). Mesenchymal Stem Cells: Cell Fate Decision to Osteoblast or Adipocyte and Application in Osteoporosis Treatment. Int J Mol Sci.

[12] Morsczeck C, Reichert TE (2018). Dental stem cells in tooth regeneration and repair in the future. Expert Opin Biol Ther.

[13] Leyendecker Junior A, Gomes Pinheiro CC, Lazzaretti Fernandes T, Franco Bueno D (2018). The use of human dental pulp stem cells for *in vivo* bone tissue engineering: A systematic review. J Tissue Eng.

[14] Aghajani F, Hooshmand T, Khanmohammadi M, Khanjani S, Edalatkhah H, Zarnani AH (2016). Comparative Immunophenotypic Characteristics, Proliferative Features, and Osteogenic Differentiation of Stem Cells Isolated from Human Permanent and Deciduous Teeth with Bone Marrow. Mol Biotechnol.

[15] Tatullo M, Marrelli M, Shakesheff KM, White LJ (2015). Dental pulp stem cells: function, isolation and applications in regenerative medicine. J Tissue Eng Regen Med.

[16] Botelho J, Cavacas MA, Machado V, Mendes JJ (2017). Dental stem cells: recent progresses in tissue engineering and regenerative medicine. Ann Med.

[17] Yamada Y, Nakamura-Yamada S, Umemura-Kubota E, Baba S (2019). Diagnostic Cytokines and Comparative Analysis Secreted from Exfoliated Deciduous Teeth, Dental Pulp, and Bone Marrow Derived Mesenchymal Stem Cells for Functional Cell-Based Therapy. Int J Mol Sci.

[18] Makino Y, Yamaza H, Akiyama K, Ma L, Hoshino Y, Nonaka K (2013). Immune therapeutic potential of stem cells from human supernumerary teeth. J Dent Res.

[19] Ding G, Niu J, Liu Y (2015). Dental pulp stem cells suppress the proliferation of lymphocytes via transforming growth factor-beta1. Hum Cell.

[20] Yamagata M, Yamamoto A, Kako E, Kaneko N, Matsubara K, Sakai K (2013). Human dental pulp-derived stem cells protect against hypoxic-ischemic brain injury in neonatal mice. Stroke.

[21] He W, Qu T, Yu Q, Wang Z, Lv H, Zhang J (2013). LPS induces IL-8 expression through TLR4, MyD88, NF-kappaB and MAPK pathways in human dental pulp stem cells. Int Endod J.

[22] Zhu L, Dissanayaka WL, Zhang C (2019). Dental pulp stem cells overexpressing stromal-derived factor-1alpha and vascular endothelial growth factor in dental pulp regeneration. Clin Oral Investig.

[23] Lambrichts I, Driesen RB, Dillen Y, Gervois P, Ratajczak J, Vangansewinkel T (2017). Dental Pulp Stem Cells: Their Potential in Reinnervation and Angiogenesis by Using Scaffolds. J Endod.

[24] Iohara K, Zheng L, Wake H, Ito M, Nabekura J, Wakita H (2008). A novel stem cell source for vasculogenesis in ischemia: subfraction of side population cells from dental pulp. Stem Cells.

[25] Hiraki T, Kunimatsu R, Nakajima K, Abe T, Yamada S, Rikitake K (2020). Stem cell-derived conditioned media from human exfoliated deciduous teeth promote bone regeneration. Oral Dis.

[26] Goldberg M, Farges JC, Lacerda-Pinheiro S, Six N, Jegat N, Decup F (2008). Inflammatory and immunological aspects of dental pulp repair. Pharmacol Res.

[27] Xu F, Qiao L, Zhao Y, Chen W, Hong S, Pan J (2019). The potential application of concentrated growth factor in pulp regeneration: an *in vitro* and *in vivo* study. Stem Cell Res Ther.

[28] Lu X, Chen X, Xing J, Lian M, Huang D, Lu Y (2019). miR-140-5p regulates the odontoblastic differentiation of dental pulp stem cells via the Wnt1/beta-catenin signaling pathway. Stem Cell Res Ther.

[29] Sun DG, Xin BC, Wu D, Zhou L, Wu HB, Gong W (2017). miR-140-5p-mediated regulation of the proliferation and differentiation of human dental pulp stem cells occurs through the lipopolysaccharide/toll-like receptor 4 signaling pathway. Eur J Oral Sci.

[30] Yang C, Jia R, Zuo Q, Zheng Y, Wu Q, Luo B (2020). microRNA-143-3p regulates odontogenic differentiation of human dental pulp stem cells through regulation of the osteoprotegerin-RANK ligand pathway by targeting RANK. Exp Physiol.

[31] Wang J, Zheng Y, Bai B, Song Y, Zheng K, Xiao J (2020). MicroRNA-125a-3p participates in odontoblastic differentiation of dental pulp stem cells by targeting Fyn. Cytotechnology.

[32] Zhang P, Yang W, Wang G, Li Y (2018). miR-143 suppresses the osteogenic differentiation of dental pulp stem cells by inactivation of NF-kappaB signaling pathway via targeting TNF-alpha. Arch Oral Biol.

[33] Qiu Z, Lin S, Hu X, Zeng J, Xiao T, Ke Z (2019). Involvement of miR-146a-5p/neurogenic locus notch homolog protein 1 in the proliferation and differentiation of STRO-1(+) human dental pulp stem cells. Eur J Oral Sci.

[34] Ji F, Pan J, Shen Z, Yang Z, Wang J, Bai X (2020). The Circular RNA circRNA124534 Promotes Osteogenic Differentiation of Human Dental Pulp Stem Cells Through Modulation of the miR-496/beta-Catenin Pathway. Front Cell Dev Biol.

[35] Yao S, Li C, Budenski AM, Li P, Ramos A, Guo S (2019). Expression of microRNAs targeting heat shock protein B8 during *in vitro* expansion of dental pulp stem cells in regulating osteogenic differentiation. Arch Oral Biol.

[36] Yang F, Huang R, Ma H, Zhao X, Wang G (2020). miRNA-411 Regulates Chondrocyte Autophagy in Osteoarthritis by Targeting Hypoxia-Inducible Factor 1 alpha (HIF-1alpha). Med Sci Monit.

[37] Wang F, Yan J (2018). MicroRNA-454 is involved in regulating trophoblast cell proliferation, apoptosis, and invasion in preeclampsia by modulating the expression of ephrin receptor B4. Biomed Pharmacother.

[38] Liu J, Ruan J, Weir MD, Ren K, Schneider A, Wang P (2019). Periodontal Bone-Ligament-Cementum Regeneration via Scaffolds and Stem Cells. Cells.

[39] Basan T, Welly D, Kriebel K, Scholz M, Brosemann A, Liese J (2017). Enhanced periodontal regeneration using collagen, stem cells or growth factors. Front Biosci (Schol Ed).

[40] Lee JS, Lee JB, Cha JK, Choi EY, Park SY, Cho KS (2017). Chemokine in inflamed periodontal tissues activates healthy periodontal-ligament stem cell migration. J Clin Periodontol.

[41] Iaculli F, Di Filippo ES, Piattelli A, Mancinelli R, Fulle S (2017). Dental pulp stem cells grown on dental implant titanium surfaces: An *in vitro* evaluation of differentiation and microRNAs expression. J Biomed Mater Res B Appl Biomater.

[42] M DEC, Radunovic M, Zizzari VL, V DIG, C DIN, Piattelli A (2018). Osteoblastic differentiating potential of dental pulp stem cells *in vitro* cultured on a chemically modified microrough titanium surface. Dent Mater J.

[43] Covarrubias C, Cadiz M, Maureira M, Celhay I, Cuadra F, von Marttens A (2018). Bionanocomposite scaffolds based on chitosan-gelatin and nanodimensional bioactive glass particles: *In vitro* properties and *in vivo* bone regeneration. J Biomater Appl.

[44] Yuan J, Liu X, Chen Y, Zhao Y, Liu P, Zhao L (2017). Effect of SOX2 on osteogenic differentiation of dental pulp stem cells. Cell Mol Biol (Noisy-le-grand).

[45] Yang X, van der Kraan PM, Bian Z, Fan M, Walboomers XF, Jansen JA (2009). Mineralized tissue formation by BMP2-transfected pulp stem cells. J Dent Res.

[46] Zhu L, Ma J, Mu R, Zhu R, Chen F, Wei X (2018). Bone morphogenetic protein 7 promotes odontogenic differentiation of dental pulp stem cells *in vitro*. Life Sci.

[47] Kaito T, Morimoto T, Mori Y, Kanayama S, Makino T, Takenaka S (2018). BMP-2/7 heterodimer strongly induces bone regeneration in the absence of increased soft tissue inflammation. Spine J.

[48] Hrubi E, Imre L, Robaszkiewicz A, Virag L, Kerenyi F, Nagy K (2018). Diverse effect of BMP-2 homodimer on mesenchymal progenitors of different origin. Hum Cell.

[49] Xia Y, Chen H, Zhang F, Bao C, Weir MD, Reynolds MA (2018). Gold nanoparticles in injectable calcium phosphate cement enhance osteogenic differentiation of human dental pulp stem cells. Nanomedicine.

[50] Paduano F, Marrelli M, White LJ, Shakesheff KM, Tatullo M (2016). Odontogenic Differentiation of Human Dental Pulp Stem Cells on Hydrogel Scaffolds Derived from Decellularized Bone Extracellular Matrix and Collagen Type I. PLoS One.

[51] Balic A (2018). Biology Explaining Tooth Repair and Regeneration: A Mini-Review. Gerontology.

[52] Yamaguchi S, Shibata R, Yamamoto N, Nishikawa M, Hibi H, Tanigawa T (2015). Dental pulp-derived stem cell conditioned medium reduces cardiac injury following ischemia-reperfusion. Sci Rep.

[53] Silva GO, Zhang Z, Cucco C, Oh M, Camargo CHR, Nor JE (2017). Lipoprotein Receptor-related Protein 6 Signaling is Necessary for Vasculogenic Differentiation of Human Dental Pulp Stem Cells. J Endod.

[54] Zhong W, Montana M, Santosa SM, Isjwara ID, Huang YH, Han KY (2018). Angiogenesis and lymphangiogenesis in corneal transplantation-A review. Surv Ophthalmol.

[55] Zhu Q, Zhu Y, Tighe S, Liu Y, Hu M (2019). Engineering of Human Corneal Endothelial Cells *In Vitro*. Int J Med Sci.

[56] Xiao L, Nasu M (2014). From regenerative dentistry to regenerative medicine: progress, challenges, and potential applications of oral stem cells. Stem Cells Cloning.

[57] Syed-Picard FN, Du Y, Lathrop KL, Mann MM, Funderburgh ML, Funderburgh JL (2015). Dental pulp stem cells: a new cellular resource for corneal stromal regeneration. Stem Cells Transl Med.

[58] Gomes JA, Geraldes Monteiro B, Melo GB, Smith RL, Cavenaghi Pereira da Silva M, Lizier NF (2010). Corneal reconstruction with tissue-engineered cell sheets composed of human immature dental pulp stem cells. Invest Ophthalmol Vis Sci.

[59] Alsaeedi HA, Koh AE, Lam C, Rashid MBA, Harun MHN, Saleh M (2019). Dental pulp stem cells therapy overcome photoreceptor cell death and protects the retina in a rat model of sodium iodate-induced retinal degeneration. J Photochem Photobiol B.

[60] Mead B, Logan A, Berry M, Leadbeater W, Scheven BA (2017). Concise Review: Dental Pulp Stem Cells: A Novel Cell Therapy for Retinal and Central Nervous System Repair. Stem Cells.

[61] Kang J, Fan W, Deng Q, He H, Huang F (2019). Stem Cells from the Apical Papilla: A Promising Source for Stem Cell-Based Therapy. Biomed Res Int.

[62] Fernandes TL, Shimomura K, Asperti A, Pinheiro CCG, Caetano HVA, Oliveira C (2018). Development of a Novel Large Animal Model to Evaluate Human Dental Pulp Stem Cells for Articular Cartilage Treatment. Stem Cell Rev Rep.

[63] Merckx G, Hosseinkhani B, Kuypers S, Deville S, Irobi J, Nelissen I (2020). Angiogenic Effects of Human Dental Pulp and Bone Marrow-Derived Mesenchymal Stromal Cells and their Extracellular Vesicles. Cells.

[64] Marchionni C, Bonsi L, Alviano F, Lanzoni G, Di Tullio A, Costa R (2009). Angiogenic potential of human dental pulp stromal (stem) cells. Int J Immunopathol Pharmacol.

[65] Janebodin K, Zeng Y, Buranaphatthana W, Ieronimakis N, Reyes M (2013). VEGFR2-dependent angiogenic capacity of pericyte-like dental pulp stem cells. J Dent Res.

[66] Nam H, Kim GH, Bae YK, Jeong DE, Joo KM, Lee K (2017). Angiogenic Capacity of Dental Pulp Stem Cell Regulated by SDF-1alpha-CXCR4 Axis. Stem Cells Int.

[67] Hilkens P, Fanton Y, Martens W, Gervois P, Struys T, Politis C (2014). Pro-angiogenic impact of dental stem cells *in vitro* and *in vivo*. Stem Cell Res.

[68] Jin R, Song G, Chai J, Gou X, Yuan G, Chen Z (2018). Effects of concentrated growth factor on proliferation, migration, and differentiation of human dental pulp stem cells *in vitro*. J Tissue Eng.

[69] Li J, Diao S, Yang H, Cao Y, Du J, Yang D (2019). IGFBP5 promotes angiogenic and neurogenic differentiation potential of dental pulp stem cells. Dev Growth Differ.

[70] Li D, Deng T, Li H, Li Y (2015). MiR-143 and miR-135 inhibitors treatment induces skeletal myogenic differentiation of human adult dental pulp stem cells. Arch Oral Biol.

[71] Martinez-Sarra E, Montori S, Gil-Recio C, Nunez-Toldra R, Costamagna D, Rotini A (2017). Human dental pulp pluripotent-like stem cells promote wound healing and muscle regeneration. Stem Cell Res Ther.

[72] Venugopal V, Pavlakis S (2021). Duchenne Muscular Dystrophy. StatPearls. Treasure Island (FL).

[73] Pisciotta A, Riccio M, Carnevale G, Lu A, De Biasi S, Gibellini L (2015). Stem cells isolated from human dental pulp and amniotic fluid improve skeletal muscle histopathology in mdx/SCID mice. Stem Cell Res Ther.

[74] Mortada I, Mortada R, Al Bazzal M (2018). Dental pulp stem cells and the management of neurological diseases: An update. J Neurosci Res.

[75] Askari N, Yaghoobi MM, Shamsara M, Esmaeili-Mahani S (2014). Human Dental Pulp Stem Cells Differentiate into Oligodendrocyte Progenitors Using the Expression of Olig2 Transcription Factor. Cells Tissues Organs.

[76] Chiu HY, Lin CH, Hsu CY, Yu J, Hsieh CH, Shyu WC (2017). IGF1R(+) Dental Pulp Stem Cells Enhanced Neuroplasticity in Hypoxia-Ischemia Model. Mol Neurobiol.

[77] Al-Zer H, Apel C, Heiland M, Friedrich RE, Jung O, Kroeger N (2015). Enrichment and Schwann Cell Differentiation of Neural Crest-derived Dental Pulp Stem Cells. *In Vivo*.

[78] Xiao L, Ide R, Saiki C, Kumazawa Y, Okamura H (2017). Human Dental Pulp Cells Differentiate toward Neuronal Cells and Promote Neuroregeneration in Adult Organotypic Hippocampal Slices *In Vitro*. Int J Mol Sci.

[79] Yamamoto A, Sakai K, Matsubara K, Kano F, Ueda M (2014). Multifaceted neuro-regenerative activities of human dental pulp stem cells for functional recovery after spinal cord injury. Neurosci Res.

[80] Hao J, Yang H, Cao Y, Zhang C, Fan Z (2020). IGFBP5 enhances the dentinogenesis potential of dental pulp stem cells via JNK and ErK signalling pathways. J Oral Rehabil.

[81] Rafiee F, Pourteymourfard-Tabrizi Z, Mahmoudian-Sani MR, Mehri-Ghahfarrokhi A, Soltani A, Hashemzadeh-Chaleshtori M (2020). Differentiation of dental pulp stem cells into neuron-like cells. Int J Neurosci.

[82] Luo L, He Y, Jin L, Zhang Y, Guastaldi FP, Albashari AA (2021). Application of bioactive hydrogels combined with dental pulp stem cells for the repair of large gap peripheral nerve injuries. Bioact Mater.

[83] Sasaki R, Matsumine H, Watanabe Y, Takeuchi Y, Yamato M, Okano T (2014). Electrophysiologic and functional evaluations of regenerated facial nerve defects with a tube containing dental pulp cells in rats. Plast Reconstr Surg.

[84] Liu AQ, Zhang LS, Fei DD, Guo H, Wu ML, Liu J (2020). Sensory nerve-deficient microenvironment impairs tooth homeostasis by inducing apoptosis of dental pulp stem cells. Cell Prolif.

[85] Ogasawara N, Kano F, Hashimoto N, Mori H, Liu Y, Xia L (2020). Factors secreted from dental pulp stem cells show multifaceted benefits for treating experimental temporomandibular joint osteoarthritis. Osteoarthritis Cartilage.

[86] Van Bellinghen X, Idoux-Gillet Y, Pugliano M, Strub M, Bornert F, Clauss F (2018). Temporomandibular Joint Regenerative Medicine. Int J Mol Sci.

[87] Zainal Ariffin SH, Kermani S, Megat Abdul Wahab R, Senafi S, Zainal Ariffin Z, Abdul Razak M (2012). *In vitro* chondrogenesis transformation study of mouse dental pulp stem cells. ScientificWorldJournal.

[88] Westin CB, Trinca RB, Zuliani C, Coimbra IB, Moraes AM (2017). Differentiation of dental pulp stem cells into chondrocytes upon culture on porous chitosan-xanthan scaffolds in the presence of kartogenin. Mater Sci Eng C Mater Biol Appl.

[89] Nemeth CL, Janebodin K, Yuan AE, Dennis JE, Reyes M, Kim DH (2014). Enhanced chondrogenic differentiation of dental pulp stem cells using nanopatterned PEG-GelMA-HA hydrogels. Tissue Eng Part A.

[90] Bousnaki M, Bakopoulou A, Papadogianni D, Barkoula NM, Alpantaki K, Kritis A (2018). Fibro/chondrogenic differentiation of dental stem cells into chitosan/alginate scaffolds towards temporomandibular joint disc regeneration. J Mater Sci Mater Med.

[91] Chamieh F, Collignon AM, Coyac BR, Lesieur J, Ribes S, Sadoine J (2016). Accelerated craniofacial bone regeneration through dense collagen gel scaffolds seeded with dental pulp stem cells. Sci Rep.

[92] Gao X, Shen Z, Guan M, Huang Q, Chen L, Qin W (2018). Immunomodulatory Role of Stem Cells from Human Exfoliated Deciduous Teeth on Periodontal Regeneration. Tissue Eng Part A.

[93] Paino F, La Noce M, Giuliani A, De Rosa A, Mazzoni S, Laino L (2017). Human DPSCs fabricate vascularized woven bone tissue: a new tool in bone tissue engineering. Clin Sci (Lond).

[94] Zheng DH, Wang XX, Ma D, Zhang LN, Qiao QF, Zhang J (2019). Erythropoietin enhances osteogenic differentiation of human periodontal ligament stem cells via Wnt/beta-catenin signaling pathway. Drug Des Devel Ther.

[95] Khorsand A, Eslaminejad MB, Arabsolghar M, Paknejad M, Ghaedi B, Rokn AR (2013). Autologous dental pulp stem cells in regeneration of defect created in canine periodontal tissue. J Oral Implantol.

[96] Lee CH, Hajibandeh J, Suzuki T, Fan A, Shang P, Mao JJ (2014). Three-dimensional printed multiphase scaffolds for regeneration of periodontium complex. Tissue Eng Part A.

[97] Aimetti M, Ferrarotti F, Cricenti L, Mariani GM, Romano F (2014). Autologous dental pulp stem cells in periodontal regeneration: a case report. Int J Periodontics Restorative Dent.

[98] Brunelli G, Motroni A, Graziano A, D'Aquino R, Zollino I, Carinci F (2013). Sinus lift tissue engineering using autologous pulp micro-grafts: A case report of bone density evaluation. J Indian Soc Periodontol.

[99] Trivedi S, Srivastava K, Saluja TS, Shyam H, Kumar S, Singh A (2020). Hydroxyapatite-collagen augments osteogenic differentiation of dental pulp stem cells. Odontology.

[100] Hokmabad VR, Davaran S, Aghazadeh M, Rahbarghazi R, Salehi R, Ramazani A (2019). Fabrication and characterization of novel ethyl cellulose-grafted-poly (varepsilon-caprolactone)/alginate nanofibrous/macroporous scaffolds incorporated with nano-hydroxyapatite for bone tissue engineering. J Biomater Appl.

[101] Huang K, Ou Q, Xie Y, Chen X, Fang Y, Huang C (2019). Halloysite Nanotube Based Scaffold for Enhanced Bone Regeneration. ACS Biomater Sci Eng.

[102] Bachhuka A, Delalat B, Ghaemi SR, Gronthos S, Voelcker NH, Vasilev K (2017). Nanotopography mediated osteogenic differentiation of human dental pulp derived stem cells. Nanoscale.

[103] Yang G, Jiang F, Lu Y, Lin S, Liu C, Li A (2021). Rapid construction and enhanced vascularization of microtissue using a magnetic control method. Biofabrication.

[104] Salgado CL, Barrias CC, Monteiro FJM (2020). Clarifying the Tooth-Derived Stem Cells Behavior in a 3D Biomimetic Scaffold for Bone Tissue Engineering Applications. Front Bioeng Biotechnol.

